# Bioimpedance analysis of fat free mass and its subcomponents and relative associations with maximal oxygen consumption in facioscapulohumeral dystrophy

**DOI:** 10.1007/s00421-024-05581-5

**Published:** 2024-08-21

**Authors:** Oscar Crisafulli, Giorgio Bottoni, Jessica Lacetera, Federico Fassio, Luca Grattarola, Emanuela Lavaselli, Giuseppe Giovanetti, Rossella Tupler, Massimo Negro, Giuseppe D’Antona

**Affiliations:** 1 CRIAMS-Sport Medicine Centre Voghera, University of Pavia, 27058 Voghera, Italy; 2 BioData Science Unit, IRCCS Mondino Foundation, 27100 Pavia, Italy; 3 Department of Public Health, Experimental and Forensic Medicine, Section of Biostatistics and Clinical Epidemiology, University of Pavia, 27100 Pavia, Italy; 4 Department of Life Sciences, University of Modena and Reggio Emilia, 41125 Modena, Italy; 5 Department of Public Health, Experimental and Forensic Medicine, University of Pavia, 27100 Pavia, Italy

**Keywords:** FSHD, ${\text{VO}}_{2} {\text{max}}$, Fat free mass, Body cell mass, Clinical stratification

## Abstract

**Purpose:**

Fat free mass (FFM) is considered the metabolically active component of human body and is positively associated with maximal oxygen uptake (${\text{VO}}_{2} {\text{max}}$). However, FFM is composed of metabolically active and inactive subcomponents whose proportion can vary depending on body composition and clinical condition, possibly affecting such association. Although it is known that in facioscapulohumeral dystrophy (FSHD) peculiar changes in body composition occur, it is unclear whether there are alterations in FFM composition and, if so, whether such alterations affect the association towards ${\text{VO}}_{2} {\text{max}}$ compared to healthy subjects (HS).

**Methods:**

To address this issue, 27 FSHD patients (mean age 37.3; 9 female) and 27 sex and age matched HS, underwent an assessment of ${\text{VO}}_{2} {\text{max}}$ by cardiopulmonary exercise tests (CPET) and body composition, with reference to FFM and its subcomponents, by bioimpedance analysis.

**Results:**

In between-groups comparison, patients showed lower amounts of body cell mass (BCM) and intracellular water (ICW) which reflect in lower BCM/FFM ratio and higher extracellular to intracellular water ratio (ECW/ICW). Patients’ ${\text{VO}}_{2} {\text{max}}$ was lower than HS and, even if with lower associative values than HS, correlated with FFM and BCM, while BCM/FFM and ECW/ICW ratios associations were observed only in HS.

**Conclusion:**

FSHD patients showed lower amount of BCM and ICW. BCM resulted as the parameter with the highest associative value with VO2max in both groups. Since ${\text{VO}}_{2} {\text{max}}$ is associated with functional ability in dystrophic patients, BCM, rather than FFM, could be an additional body composition-based clinical stratification factor.

## Introduction

Facioscapulohumeral dystrophy (FSHD) is the third most common muscular dystrophy, with an estimated prevalence of between 1: 15,000 and 1: 20,000 (Hamel et al. 2019). Subjects with FSHD, undergo peculiar changes in body composition which involves a gradual loss of fat free mass (FFM) and an increase in fat mass (FM) (Skalsky et al. 2008; Vera et al. 2022b).

In healthy subjects (HS), FFM is positively associated with ${\text{VO}}_{2} {\text{max}}$ (Hunt et al. 1998; Amara et al. 2001) which represents the maximal oxygen uptake (Zeiher et al. 2019) and is considered an indicator of the ability to sustain exertion in both exercise and daily physical activities (Jouannot 2001). However, FFM is composed of both metabolically active and non-active subcomponents that can quantitatively change their ratio depending on subject’s age, body composition, and clinical condition (Waki et al. 1991; Gallagher et al. 1996; Sergi et al. 2004), thus suggesting that, as its composition varies, FFM may not be a reliable indicator of ${\text{VO}}_{2} {\text{max}}$. Specifically, FFM subcomponents are body cell mass (BCM), which is the only metabolically active FFM component (Wang et al. 2007), extracellular mass (ECM), and total body water (TBW) (Kasugai 2002), which in turn is separable into intracellular (ICW) and extracellular water (ECW). Notably, BCM and ECM are cellular elements, while FFM, TBW, ICW and ECW are molecular elements (Paoli and Campa 2024). In HS, the relationship between maximal oxygen consumption and FFM, as well as its subcomponents and their quantitative ratio, has been considered in many studies. For instance, in a study which analyzed a sample of 3848 HS (Köhler et al. 2018), BCM, showed a stronger association with ${\text{VO}}_{2} {\text{peak}}$ than FFM, confirming previous data reported in a sample of 56 adult HS (Chen et al. 2009). Moreover, a recent work by Yamada et al. (2023) reported that, in a sample of 115 adult HS, BCM/FFM and ECW/ICW ratios were significant predictors of ${\text{VO}}_{2} {\text{peak}}$.

The common consideration of FFM as the metabolically active part of the body presupposes the maintenance of a relatively constant relationship with BCM (Gallagher et al. 1996) but this aspect has never been investigated in FSHD. The issue arises as a clinical condition that causes peculiar changes in body composition, like FSHD, could alter FFM composition, possibly altering the associations observed in HS. To date, only one work has reported an association between ${\text{VO}}_{2} {\text{peak}}$ and body composition in a patient’s sample (Vera et al. 2022a), suggesting that the disease driven FFM loss contributes to the lower VO2peak observed in a group of 11 adult patients compared to HS. However, several points still need to be addressed. For example, although it is known that FSHD involves a gradual loss of FFM, it is not known whether this reduction affects all its components equally or whether there are alterations in only some of them. Consequently, it is unclear whether BCM/FFM and ECW/ICW ratios are comparable to those of HS and if they have the same associative values with ${\text{VO}}_{2} {\text{max}}$. Furthermore, it is unclear whether the metabolically active residual mass (whether expressed as FFM or BCM) has a significant level of association with ${\text{VO}}_{2} {\text{max}}$. In fact, considering the mitochondrial impairment (Slipetz et al. 1991) and the presence of muscle damage from oxidative stress peculiar of FSHD (Heher et al. 2022), it seems possible that patient’s residual metabolically active mass may become dysfunctional, possibly compromising the association. Since ${\text{VO}}_{2} {\text{max}}$ can provide important indications about physical efficiency in patients affected by neuromuscular diseases (Markvardsen et al. 2019) and muscular dystrophies (Sveen et al. 2008; Bankolè et al. 2016), shedding light on these aspects could provide not only important indications about the effects of the disease on both structural and functional domains, but also further elements of clinical stratification, aspect of major importance for defining adequate treatments (Salsi et al. 2023).

Hence, in the present study we used a safe and non-invasive technique (bioimpedance analysis (BIA)), previously used in other dystrophic conditions (Duchenne, myotonic type 1, and congenital muscular dystrophies) (Schwartz et al. 2016; Vermeulen et al. 2019; Nicholsok et al. 2018, Grilo et al. 2020; Saure et al. 2018; Mok et al. 2006; Mok et al. 2010; Rinninella et al. 2019), to evaluate FFM and its subcomponents in 27 FSHD subjects, investigating their degree of association with ${\text{VO}}_{2} {\text{max}}$ and comparing the data with a cohort of sex and age matched HS.

## Materials and methods

### Participants

The study was carried out at the CRIAMS-Sport Medicine Centre, Voghera. Participants, consecutively recruited, were enrolled in the study if they met the following inclusion criteria: clinical or genetic diagnosis of FSHD; enrolment in the Italian National Register for FSHD. Exclusion criteria were wheelchair bound at selection; use of corticosteroids; cardiac and respiratory dysfunctions; psychological or psychiatric disorders; major osteoarticular dysfunctions. Patients were assigned to the four clinical categories of the Complete Clinical Evaluation form (Ricci et al. 2016), which classifies subjects based on: facial and scapular girdle muscle weakness (category A); muscle weakness limited to the scapular girdle or facial muscles (category B); no symptoms (category C); and myopathic phenotype presenting inconsistent clinical features with the canonical FSHD phenotype (category D). Additionally, a sample of sex and age matched healthy subjects was also enrolled as control group**.** All participants provided written, informed consent to participate in this study, which was conducted according to the Declaration of Helsinki. The study was approved by Lombardy Territorial Ethics Committee 6, protocol number 0006176/24.

### Body composition assessment

Weight and height parameters were obtained from all participants and Body Mass Index (BMI) was then calculated as body mass (kg) divided by height’s square (m). Body composition analysis was performed using a bioelectrical impedance analysis (BIA EFG, Akern, Florence, Italy) following the manufacturer’s instructions (e.g., subjects lying supine with limbs extended away from the trunk). The electrodes (BIATRODES, Akern, Florence, Italy) were conventionally placed on the right side of the body (metacarpal and wrist lines; lines metatarsals and ankle). The parameters considered were FFM and its subcomponents, i.e., BCM, ECM, ICW, ECW and TBW. The BCM/FFM and ECW/ICW ratio were then calculated. The estimates were obtained with the Bodygram PRO v.3.0 software. To estimate FFM in the age range of 10 to 16 years, the software employs proprietary clinically validated equations (Vicente-Rodriguez et al. 2011) while, in the adults and geriatric populations, the equation for estimating FFM is from Sun et al. (2003). Regarding BCM, the software implements the equation ${\text{BCM }} = \, 0.29 \, *{\text{ FFM }}*{\text{ Ln }}*{\text{ Pha}}$, which has been clinically validated in Kotler et al. (1996), and Kotler et al. (1999). Based on these estimates, ECM is obtained by subtracting BCM from FFM. The equations of TBW and ECW are clinically validated by Ward et al. (2001) and Biasioli et al. (2001), respectively. Finally, ICW is obtained by subtracting ECW from TBW.

### Maximal oxygen consumption assessment

All participants performed a maximal incremental exercise on a cycle ergometer (E 100, Cosmed, Italy) under electrocardiographic guidance, supervised by a medical doctor (GD), to check for cardiac events. During the tests, pulmonary gas exchange was measured breath-by-breath using a face mask (${\text{V}}_{2}$ Mask TM, Hans Rudolph Inc.), connected to a gas analyser (Quark PFT, Cosmed, Italy). The test was performed with the step incremental technique (15 Watt (W) every 30 s (s), with a previous warm-up of 3.5 min (min) at 25 W) (Parimbelli et al. 2021). ${\text{VO}}_{2} {\text{max}}$ and respiratory exchange ratio (RER) were determined. The test was considered maximal when it met three criteria: RER > 1.1, ratio of perceived exertion (RPE) ≥ 8 and ${\text{VO}}_{2}$ slope at plateau for at least 30 s. RER was derived from raw value, while VO2max was calculated as the average of the 30 s following the achievement of RER = 1.1 and RPE ≥ 8. For each patient, the test was performed at 11 a.m. in a room with a constant temperature of 23 °C.

### Statistical analysis

Categorical variables are described as count and percentages, while continuous variables as mean and standard deviation (SD) and median and 1st–3rd quantile (with minimum and maximum value). Variables distribution was explored both with Shapiro–Wilk’s test and QQ-plots representation. Comparisons between FSHD patients and HS were performed with Chi-square test (Yates’ continuity correction) and Student’s *t*-test (and Mann–Whitney *U* test as validation), according to their nature. The difference between groups was also described in terms of effect size, due to the small sample size: phi coefficient for categorical variables was estimated while Cohen’s *d* for continuous ones (and Wilcoxon effect size as validation, estimated as z-score/√n). Since most of variables are normally distributed and linearly associated, we performed a Pearson’s correlation coefficient estimation and related significance test, in which both ${\text{VO}}_{2} {\text{max}}$ and ${\text{VO}}_{2} {\text{max}}$/weight were assumed as dependent variables. The ${\text{VO}}_{2} {\text{max}}$ parameter in FSHD subjects included an outlier value (> 4000 ml/min): correlation analyses were conducted after excluding this subject since plot of the residuals of the regression models confirmed its outlier status. Similarly, another FSHD subject had outlier values in the BCM/FFM (0.23) and ECW/ICW (2.86) ratios, therefore, in the results section, the correlation analysis between ${\text{VO}}_{2} {\text{max}}$ and such parameters are reported both with and without this subject. The significance level was set as 0.05. The analysis was conducted with R software v.4.2.3.

## Results

### Participants

Participant characteristics are summarized in Table 1. 54 subjects participated in the study, 27 FSHD patients (9 females; mean age 37.3 ± 13.8; range 16–61) and 27 HS (10 females; mean age 35.5 ± 14.7; range 17–66). Among FSHD cohort, 17 patients belong to clinical category A, 7 to clinical category B, 1 to clinical category C and 2 to clinical category D. No significant differences were found between groups for age (*p* = 0.65) and gender (*p* > 0.90).

**Table 1:** Description of study sample and comparison among the two groups

	FSHD (*N* = 27)	HS (*N* = 27)	*p*-value	Effect Size	Overall (*N* = 54)
Gender					
Female	9 (33.3%)	10 (37.0%)	> 0.90^a^	0.04*	19 (35.2%)
Male	18 (66.7%)	17 (63.0%)			35 (64.8%)
Age (years)					
Mean (sd)	37.3 (13.9)	35.5 (14.7)	0.650^b^	0.12**	36.4 (14.2)
Median [Q1; Q3]	38.0 [27.0; 47.0]	37.0 [20.5; 46.0]	0.723^c^	0.05***	37.5 [23.0; 46.0]
Min; Max	16.0; 61.0	17.0; 66.0			16.0; 66.0
Height (cm)					
Mean (sd)	173.2 (7.3)	171.9 (9.6)	0.554^b^	0.16**	172.5 (8.4)
Median [Q1; Q3]	174.0 [166.9; 178.0]	173 [165.0; 180.0]	0.646^c^	0.06***	173 [166.0; 179.0]
Min; Max	157.0; 188.0	156.0; 188.0			156.0; 188.0
Weight (kg)					
Mean (sd)	71.3 (13.8)	72.6 (13.4)	0.733^b^	0.09**	71.9 (13.5)
Median [Q1; Q3]	67.0 [63.6; 75.9]	73.0 [62.0; 82.7]	0.562^c^	0.08***	69.8 [62.8; 82.2]
Min; Max	46.8; 103.1	44.7; 92.5			44.7; 103.1
BMI (kg/m^2^)					
Mean (sd)	23.7 (3.5)	24.4 (2.7)	0.410^b^	0.23**	24.0 (3.1)
Median [Q1; Q3]	23.4 [21.3; 24.8]	24.6 [23.3; 26.1]	0.104^c^	0.22***	24.1 [22.0; 25.8]
Min; Max	18.7; 33.7	17.5; 29.4			17.5; 33.7
$${\text{VO}}_{2} {\text{max}}$$(ml/min)					
Mean (sd)	2034.6 (643.9)	2596.6 (778.4)	0.006^b^	0.79**	2315.6 (762.2)
Median [Q1; Q3]	1946.0 [1621.5; 2293.5]	2668.0 [1971.5; 3116.0]	0.005^c^	0.38***	2220.0 [1694.5; 2703.3]
Min; Max	1145.0; 4415.0	1301.0; 4132.0			1145.0; 4415.0
${\text{VO}}_{2} {\text{max}}$/weight (ml/min/kg)					
Mean (sd)	29.0 (8.8)	35.8 (8.6)	0.006^b^	0.78**	32.4 (9.3)
Median [Q1; Q3]	28.4 [22.1; 32.5]	34.7 [29.6; 41.4]	0.002^c^	0.42***	31.1 [25.0; 36.9]
Min; Max	19.6; 58.2	22.2; 56.6			19.6; 58.2
FFM (kg)					
Mean (sd)	54.2 (11.3)	57.7 (11.1)	0.260^b^	0.31**	56.0 (11.2)
Median [Q1; Q3]	52.0 [46.4; 58.8]	60.8 [48.5; 65.7]	0.174^c^	0.19***	55.7 [46.9; 64.8]
Min; Max	37.1; 79.3	38.8; 74.4			37.1; 79.3
BCM (kg)					
Mean (sd)	28.2 (8.56)	33.9 (7.86)	0.013^b^	0.70**	31.1 (8.65)
Median [Q1; Q3]	26.7 [22.4; 32.0]	34.5 [28.6; 39.5]	0.016^c^	0.33***	31.0 [24.0; 38.2]
Min; Max	13.3; 45.3	20.7; 47.8			13.3; 47.8
BCM/FFM					
Mean (sd)	0.51 (0.1)	0.58 (0.0)	< 0.001^b^	1.16**	0.5 (0.1)
Median [Q1; Q3]	0.3 [0.5; 0.6]	0.6 [0.6; 0.6]	< 0.001^c^	0.54***	0.6 [0.5; 0.6]
Min; Max	0.2; 0.6	0.5; 0.7			0.2; 0.7
ECM (kg)					
Mean (sd)	26.1 (5.7)	23.8 (3.7)	0.082^b^	0.48**	24.9 (4.9)
Median [Q1; Q3]	25.0 [22.5; 28.6]	24.0 [20.5; 26.8]	0.177^c^	0.19***	24.3 [21.8; 27.3]
Min; Max	18.5; 43.7	17.0; 29.8			17.0; 43.7
ECW (lt)					
Mean (sd)	19.0 (4.0)	17.3 (2.8)	0.092^b^	0.47**	18.1 (3.5)
Median [Q1; Q3]	18.1 [16.4; 21.4]	17.6 [14.9; 19.7]	0.210^c^	0.17***	17.7 [15.9; 19.9]
Min; Max	13.4; 30.9	12.3; 21.8			12.3; 30.9
ICW (lt)					
Mean (sd)	21.0 (6.2)	24.8 (5.9)	0.023^b^	0.64**	22.9 (6.3)
Median [Q1; Q3]	19.9 [16.8; 24.2]	25.2 [20.7; 28.7]	0.027^c^	0.30***	22.3 [17.8; 28.1]
Min; Max	10.8; 33.3	15.0; 35.6			10.8; 35.6
ECW/ICW					
Mean (sd)	1.0 (0.4)	0.7 (0.1)	0.004^b^	0.84**	0.8 (0.3)
Median [Q1; Q3]	0.9 [0.8; 1.0]	0.7 [0.7; 0.8]	< 0.001^c^	0.52***	0.8 [0.7; 0.9]
Min; Max	0.6; 2.9	0.6; 0.9			0.6; 2.9
TBW (lt)					
Mean (sd)	40.0 (8.3)	42.1 (8.2)	0.340^b^	0.26**	41.0 (8.2)
Median [Q1; Q3]	38.1 [34.0; 45.0]	44.7 [35.4; 47.9]	0.243^c^	0.16***	40.8 [34.4; 47.6]
Min; Max	27.1; 57.9	27.7; 54.4			27.1; 57.9

^*^Phi coefficient; **Cohen’s d; ***Wilcoxon effect size. ^a^Chi-square (Yates’ correction); ^b^Student’s t-test; ^c^ Mann–Whitney *U* test

*FSHD* facioscapulohumeral dystrophy, *HS* Healthy subjects, *sd* standard deviation, *Q* quantile, *Min* minimum, *Max*, maximum, cm, centimetres, *kg* kilograms, *BMI* body mass index, *m* meters, *ml* millilitres, *lt* litres, *min* minute, *FFM* free fat mass, *BCM* body cell mass, *FM* fat mass, *ECM* extra cellular mass, *ECW* extra cellular water, *ICW* intra cellular water, *TBW* total body water

### Body composition comparison

The results of the comparison analysis are reported in Table 1. The two groups were similar for weight (*p* = 0.733), height (*p* = 0.554), BMI (*p* = 0.410), FFM (*p* = 0.260), ECM (*p* = 0.082), ECW (*p* = 0.092) and TBW (*p* = 0.340). Instead, FSHD patients show significantly lower values of BCM (*p* = 0.013), BCM/FFM ratio (*p* < 0.001), ICW (*p* = 0.023) and a significantly higher ECW/ICW ratio (*p* = 0.004).

### Maximal oxygen consumption and fat free mass parameters associations

FSHD patients’ VO2max resulted significantly lower than HS as both absolute value (*p* = 0.006) and normalized for body weight (*p* = 0.006) (see Table 1). Table 2 reports the assessment of the correlation analysis between VO2max and FFM, BCM, BCM/FFM and ECW/ICW for both FSHD and HS. When ${\text{VO}}_{2} {\text{max}}$ is expressed as absolute value, both groups show a significant correlation with FFM (FSHD: Pearson’s ρ = 0.48, *p* = 0.014; HS: Pearson’s ρ = 0.72, *p* < 0.001) and BCM (FSHD: Pearson’s ρ = 0.49, *p* = 0.011; HS: Pearson’s ρ = 0.75, *p* < 0.001). Instead, HS, unlike FSHD, report significant correlations also with BCM/FFM ratio (Pearson’s ρ = 0.67, *p* < 0.001), and ECW/ICW ratio (Pearson’s ρ =  – 0.69, *p* < 0.001). When the FSHD outlier is removed from the sample (N = 25), the correlation values increase for both BCM/FFM (Pearson’s ρ = 0.39, *p* = 0.054) and ECW/ICW (Pearson’s ρ = – 0.37, *p* = 0.067). When VO2max is expressed as normalized for body weight, only HS report significant correlations with BCM/FFM (Pearson’s ρ = 0.50, *p* = 0.009) and ECW/ICW ratios (Pearson’s ρ =  – 0.51, *p* = 0.007). In this last analysis, the removal of the FSHD outlier leaves the correlation values almost equal to those observed on the 26 subjects for both BCM/FFM (Pearson’s ρ = 0.16, *p* = 0.450) and ECW/ICW (Pearson’s ρ =  – 0.17, *p* = 0.409).

## Discussion

In this study, the analysis of FSHD patients’ FFM composition, VO2max, and relative associations has been performed for the first time. The comparison of the obtained values with those of a control sample highlighted several structural and functional differences.

### Structural domain comparison

Our results show that, notwithstanding the similarities for most of the anthropometrics and body composition parameters (see Table 1), FSHD patients have less BCM and ICW, which results in the alteration of the BCM/FFM, and ECW/ICW ratios, respectively. The lower amount of BCM compared to HS is in line with what has already been reported in a population of 43 patients suffering from Duchenne muscular dystrophy (Vermeulen et al. 2019). Since BCM is typically calculated on the estimate of ICW (De Lorenzo et al. 1997; Earthman et al. 2007), the concomitant decrease of the two parameters seems to be the expression of the same pathological phenomenon. The patient’s BCM/FFM ratio results significantly lower compared to controls, whose observed mean value matches the one reported in the literature as reference for HS (0.58) (Wang et al. 2007; Yamada et al. 2023). On the other hand, the higher ECW/ICW ratio, due to the link between ICW and BCM, would be a further indicator of the metabolically active quote reduction. This would be coherent with previous data in which the augmentation of such ratio was linked to physical worsening manifestations like muscle attenuation (Ohashi et al. 2018), decreased handgrip strength and slower gait (Hioka et al. 2021). It remains unclear why the TBW levels remain comparable despite the significant difference in ICW. However, a possible explanation can be drawn from the observation of the data which shows that, compared to HS, the patients’ lower value of ICW is accompanied by a higher value of ECW (Table 1), although such majority doesn’t reach statistical significance. Interestingly, a high ECW value has been proposed as an inflammation marker (Geronikolou et al. 2017) which is a common tract of FSHD (Wang and Tawil 2016; Denny and Heather 2017; Greco et al. 2024). Therefore, although we can’t state this with certainty, having not analyzed inflammatory biomarkers, it seems possible that the similarity of TBW reflects two distinct pathological phenomena, the reduction of BCM (and, therefore, of ICW) and the manifestation of an inflammatory state.

### Functional domain comparison

**Fig. 1:**
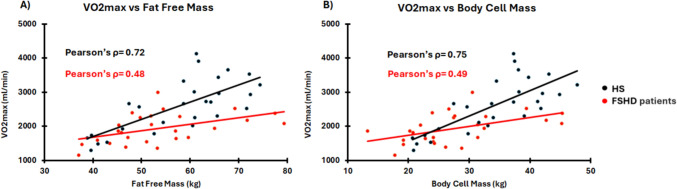
Graphical representation of the correlation between ${\text{VO}}_{2} {\text{max}}$ and FFM (panel A) and BCM (panel B) for both groups. The red graphic elements refer to FSHD patients (*N* = 26) while the black elements refer to HS (*N* = 27)

In line with a previous study (Vera et al. 2022a), ${\text{VO}}_{2} {\text{max}}$ of FSHD patients resulted significantly lower than HS, both as absolute and normalized values (Table 1). For both groups, the absolute ${\text{VO}}_{2} {\text{max}}$ was associated with FFM. Moreover, in line with previously reported data on HS (Köhler et al. 2018; Chen et al. 2009), the association with BCM resulted stronger than the one obtained with FFM for both groups. Interestingly, although such associations reach significance also in the patient group, their values are lower compared to HS (Fig. 1). If, on one hand, the lower association of FFM vs VO2max observed in the patients could be due to the lower observed amount of metabolically active quote (Table 1), it remains unclear why BCM has also a lower level of association with such parameter. However, it seems possible that the mitochondrial involvement (decreased cytochrome c oxidase activity and reduced ATP synthesis) and oxidative stress damage reported in FSHD and contributing to progressive muscular dysfunction (Turki et al. 2012; Celegato et al. 2006), may concur to the observed weaker association of BCM with ${\text{VO}}_{2} {\text{max}}$. If this was the case, both quantity and quality of BCM would impact on the observed lower FFM vs ${\text{VO}}_{2} {\text{max}}$ association (Table 2).

**Table 2:** ${\text{VO}}_{2} {\text{max}}$ and ${\text{VO}}_{2} {\text{max}}$/Weight correlations

		FSHD (*N* = 26)		Healthy (*N* = 27)	
		Pearson’s ρ	*p*-value	Pearson’s ρ	*p*-value
${\text{VO}}_{2} {\text{max}}$(ml/min)	FFM (kg)	0.48	0.014	0.72	< 0.001
${\text{VO}}_{2} {\text{max}}$(ml/min)	BCM (kg)	0.49	0.011	0.75	< 0.001
${\text{VO}}_{2} {\text{max}}$(ml/min)	BCM/FFM	0.29	0.153	0.67	< 0.001
${\text{VO}}_{2} {\text{max}}$(ml/min)	ECW/ICW	– 0.19	0.343	– 0.69	< 0.001
${\text{VO}}_{2} {\text{max}}$/weight (ml/min)/Kg	FFM (kg)	– 0.22	0.280	0.24	0.238
${\text{VO}}_{2} {\text{max}}$/weight (ml/min)/Kg	BCM (kg)	– 0.07	0.737	0.31	0.120
${\text{VO}}_{2} {\text{max}}$/weight (ml/min)/Kg	BCM/FFM	0.18	0.379	0.50	0.009
${\text{VO}}_{2} {\text{max}}$/weight (ml/min)/Kg	ECW/ICW	– 0.17	0.421	– 0.51	0.007

*FSHD* facioscapulohumeral dystrophy, *ml* millilitres, *min* minute, *FFM* free fat mass, kilograms, *BCM* body cell mass, *ECW* extra cellular water, *ICW* intra cellular water

Coherently with a previous article (Yamada et al. 2023), the BCM/FFM ratio was significantly associated with both absolute and normalized ${\text{VO}}_{2} {\text{max}}$ in HS, but not in FSHD patients, possibly because of the ratio alteration due to BCM loss. Besides, the ECW/ICW ratio was negatively associated with both absolute and normalized ${\text{VO}}_{2} {\text{max}}$ in HS, but not in FSHD patients, speculatively because of the abnormal fluid distribution dictated by metabolically active quote loss and, possibly, inflammatory state. Such associations in FSHD subjects remain nonsignificant even after removal of the outlier, which would be confirmative of the reported considerations. However, the approach to the significance level observed in the correlation analysis with the absolute ${\text{VO}}_{2} {\text{max}}$ values, underlines the need of further investigations on a larger sample of patients.

Overall, all data concord to indicate BCM as a fundamental determinant of FFM composition and may be considered within the factors contributing to VO2max in both HS and FSHD patients.

### Limitations and future perspectives

The present work suffers some limitations. From a recent systematic review, emerges the scarcity of equations for estimating BCM (Campa et al. 2024); thus, it seems conceivable that, in the future, these equations will neither be improved, nor new ones presented. Since the reference method for measuring BCM (whole-body counting of ^40^ K; Pierson and Wang 1988) implies high costs and restricted availability (Dittmar and Reber 2004), the search for alternative solutions for estimating this parameter will be of major importance. Besides, further investigations on wider samples must be conducted to confirm or disprove the present results. The sample size does not allow to support a multivariable regression analysis to evaluate the influence of possible confounders, such as gender or age, on the identified associations. Moreover, due to sample dimension, we were not able to assess eventual differences in FFM composition, ${\text{VO}}_{2} {\text{max}}$ and relative associations between FSHD clinical subcategories. Moreover, it remains unclear whether differences in physical exercise and/or daily physical activity may have a direct effect on BCM, thus impacting on the observed levels of association with the aerobic fitness. For instance, we cannot exclude that this confounder could have had an impact on the higher ${\text{VO}}_{2} {\text{max}}$ value observed in the FSHD outlier. However, the present findings open the path to several research perspectives. For instance, future studies should address whether, in a group of category C patients, which are clinically asymptomatic, the associations analyzed in the present work are similar or different to those of HS; the identification of eventual differences may open the path to the search for metabolic alterations not yet described in FSHD. Moreover, the results suggest that BCM could also be useful in monitoring the temporal trend of the disease (through repeated measures over time), or as a litmus test to evaluate the effectiveness of interventions aimed at improving/maintaining physical efficiency (such as adapted physical exercise programs). Shedding light on these aspects could bring significant improvements to the clinical management of FSHD.

## Conclusions

Our results indicate that, among the anthropometric measures, FFM may not be a reliable indicator of physical efficiency in FSHD patients as, compared to HS, its metabolically active quote is significantly lower. Given the observed higher association value with ${\text{VO}}_{2} {\text{max}}$, BCM arises as the more reliable anthropometric indicator of patient’s physical condition. These results could be considered to provide an additional stratification factor according to patient’s body composition. Future studies should clarify whether changes in the level of physical activity and exercise could positively act on quantitative and or/ qualitative impairments of patient’s BCM.

## Data Availability

The data that support the findings of this study are available from the corresponding author upon reasonable request.

## Abbreviations

ATP: Adenosine triphosphate
BCM: Body cell mass
BMI: Body mass index
$${\text{CO}}_{2}$$: Carbon dioxide
CPET: Cardiopulmonary exercise tests
ECM: Extracellular mass
ECW: Extracellular water
FFM: Fat free mass
FM: Fat mass
FSHD: Facioscapulohumeral dystrophy
ICW: Intracellular water
kg: Kilograms
m: Meters
RER: Respiratory exchange ratio
RPE: Ratio of perceived exertion
SD: Standard deviation
TBW: Total body water
${\text{VCO}}_{2}$: Carbon dioxide production
${\text{VE/VCO}}_{2}$: Ventilatory equivalent for CO2
$${\text{VO}}_{2}$$: Oxygen consumption
${\text{VO}}_{2} {\text{max}}$: Maximal oxygen uptake
W: Watt
